# Myeloma and *Cystoisospora* belli

**DOI:** 10.4274/tjh.galenos.2021.2021.0356

**Published:** 2021-08-25

**Authors:** Pathum Sookaromdee, Viroj Wiwanitkit

**Affiliations:** 1 Private Academic Consultant, Bangkok, Thailand; 2 Honorary Professor, Dr. D.Y. Patil University, Pune, India

**Keywords:** Blood, Cancer, Myeloma, Cystoisospora

## To the Editor,

We would like to share our ideas on “Prolonged Severe Watery Diarrhea in a Long-Term Myeloma Survivor: An Unforeseen Infection with *Cystoisospora belli*” regarding multiple myeloma (MM) patients [1]. Tiryaki et al. [1] concluded that “Parasitic infections are very uncommon… In MM diarrhea points mainly to infection in acute or chronic form,” further noting that, to their best knowledge, “this [was] the first case of a patient with MM with *C. belli* infection” [1]. The incidence of parasitic infection is usually associated with local geography. In developing countries without good hygienic foundations, parasitic infections are common but there is usually no routine screening of MM patients. In a recent report from Brazil, de Castro et al. [2] studied infectious diarrhea in autologous stem cell transplantation cases, including myeloma patients, and found that there were many parasitic infections including *C. belli* infections. In conclusion, we suggest a new recommendation for screening for parasitic infection in any patients with MM and other hematological malignancies.
