# Supplementary material for: Myeloma and Cystoisospora belli
Source: Turk J Haematol. 2021 Aug 25;38(3):229–30. doi: 10.4274/tjh.galenos.2021.2021.0356 (PMC8386304; doi:10.4274/tjh.galenos.2021.2021.0356)
Supplement: Related texts

# REPLY FROM THE AUTHORS

We have reviewed the commentary by Sookaromdee and Wiwanitkit and our reply is as follows.

We observed a patient with long-term multiple myeloma (MM) and an infrequent infection with *Cystoisospora belli* [1]. An intestinal-type chronic diarrhea was the only symptom contributing to the diagnosis. We wished to emphasize awareness of *C. belli* infection in MM patients. Sookaromdee and Wiwanitkit are of the same opinion as they mentioned the importance of *C. belli* infection in MM patients and emphasized that recommendations should be developed based on regional characteristics, especially in relation to poor hygiene conditions. They also cited an original study reported from Brazil to demonstrate that the infection could be observed in this patient population [2]. The mentioned study included 47 hematological malignancy patients and evaluated the infectious complications in patients who underwent autologous stem cell transplantation. The number of MM patients was 29. The study did not specify infectious agents according to primary diagnosis. The only result given in that study that might be pertinent for *C. belli* infection was that the authors observed 7 coccidia cases. Coccidia subgroups were not defined. For that reason, we did not cite that study in our original manuscript. Indeed, our aim was similar to that of Sookaromdee and Wiwanitkit; when treating MM patients from variable hygienic backgrounds with the complication of diarrhea, *C. belli* should be considered. Thus, the letter above supports our original argument.

**Tarık Onur Tiryaki, Kadir Uluç Anıl, Melek Büyük, Ahmet Yasir Yıldırım, Alp Atasoy, Aslı Çiftçibaşı Örmeci, Sevgi Kalayoğlu Beşışık**

## References

[1] Tiryaki TO, Anıl KU, Büyük M, Yıldırım AY, Atasoy A, Örmeci AC, Beşışık SK (2021). Prolonged severe watery diarrhea in a long-term myeloma survivor: an unforeseen infection with Cystoisospora belli. Turk J Hematol.

[2] de Castro MD, Chebli JM, Costa LJ, Alves KRL, Atalla A, Neto AEH (2018). Infectious diarrhea in autologous stem cell transplantation: high prevalence of coccidia in a South American center. Hematol Transfus Cell Ther.
